# Supplementary material for: A gene signature related to programmed cell death to predict immunotherapy response and prognosis in colon adenocarcinoma
Source: PeerJ. 2025 Feb 10;13:e18895. doi: 10.7717/peerj.18895 (PMC11823652; doi:10.7717/peerj.18895)
Supplement: Supplemental Information 1 [file peerj-13-18895-s001.docx]

**Supplementary Table 1:** Information on clinical samples of COAD patients obtained based on TCGA.

| **Characteristics** | **TCGA(N=427)** |
| --- | --- |
| **Age** |  |
| Mean±SD | 66.51±13.00 |
| Median[min-max] | 68.00[31.00,90.00] |
| **Gender** |  |
| FEMALE | 197(46.14%) |
| MALE | 230(53.86%) |
| **pathologic_T** |  |
| T1 | 10(2.34%) |
| T2 | 73(17.10%) |
| T3 | 293(68.62%) |
| T4 | 50(11.71%) |
| unknown | 1(0.23%) |
| **pathologic_N** |  |
| N0 | 248(58.08%) |
| N1 | 103(24.12%) |
| N2 | 76(17.80%) |
| **pathologic_M** |  |
| M0 | 315(73.77%) |
| M1 | 60(14.05%) |
| unknown | 52(12.18%) |
| **pathologic_stage** |  |
| I | 70(16.39%) |
| II | 163(38.17%) |
| III | 124(29.04%) |
| IV | 60(14.05%) |
| unknown | 10(2.34%) |
| **OS** |  |
| Alive | 331(77.52%) |
| Death | 96(22.48%) |
| **OS.time** |  |
| Mean±SD | 908.87±776.22 |
| Median[min-max] | 718.00[31.00,4502.00] |
