# Supplementary material for: A gene signature related to programmed cell death to predict immunotherapy response and prognosis in colon adenocarcinoma
Source: PeerJ. 2025 Feb 10;13:e18895. doi: 10.7717/peerj.18895 (PMC11823652; doi:10.7717/peerj.18895)
Supplement: Supplemental Information 2 [file peerj-13-18895-s002.docx]

**Supplementary Table 2: Sequence of target gene primer pairs for RT-qPCR.**

| **Gene** | **Forward primer sequence (5’-3’)** | **Reverse primer sequence (5’-3’)** |
| --- | --- | --- |
| HSPA1A | TCCGCAGCACGTTGAGCC | CAAGATGAAGGAGATCGCCG |
| SEMA4C | ACTGAGTGTATCCAGAAAGG | CTCCCTGAAGAAGAAGTAGA |
| ARHGAP4 | TAACTACTACCTGCATGACG | CTGGATAAACTTCTCCATGT |
| CDKN2A | CCTCCCGGGCAGCGTCGT | ATAGTTACGGTCGGAGGCCG |
| ATOH1 | ATTGCGCAGCTGGTCGAA | GGGGTGGTGGTAGACGAGCT |
| ZG16 | GATCTCCTCCAGGTCTCCGTTG | AGTTGGACGGCCCCATCA |
| GAPDH | GAAGGCTGGGGCTCATTT | CAGGAGGCATTGCTGATGAT |
